# A Sustainable Route to Ruthenium Phosphide (RuP)/Ru Heterostructures with Electron‐Shuttling of Interfacial Ru for Efficient Hydrogen Evolution

**DOI:** 10.1002/advs.202309869

**Published:** 2024-03-28

**Authors:** Daohao Li, Rongsheng Cai, Dongyong Zheng, Jun Ren, Chung‐Li Dong, Yu‐Cheng Huang, Sarah J. Haigh, Xien Liu, Feilong Gong, Yiming Liu, Jian Liu, Dongjiang Yang

**Affiliations:** ^1^ State Key Laboratory of Bio‐fibers and Eco‐textiles College of Materials Science and Engineering School of Environmental Science and Engineering Institute of Marine Biobased Materials Qingdao University Qingdao 266071 P. R. China; ^2^ Department of Materials University of Manchester Manchester M13 9PL UK; ^3^ School of Chemical and Environmental Engineering North University of China Taiyuan 030051 P. R. China; ^4^ Department of Physics Tamkang University New Taipei City 25137 Taiwan; ^5^ State Key Laboratory Base of Eco‐Chemical Engineering College of Chemical Engineering Qingdao University of Science and Technology Qingdao 266042 P. R. China; ^6^ Key Laboratory of Surface and Interface Science and Technology of Henan Province College of Material and Chemical Engineering Zhengzhou University of Light Industry Zhengzhou Henan 450001 P. R. China; ^7^ College of Materials Science and Engineering Taiyuan University of Technology Taiyuan 030024 P. R. China; ^8^ Science Center of Energy Material and Chemistry, College of Chemistry and Chemical Engineering Inner Mongolia University Hohhot 010021 P. R. China; ^9^ DICP‐Surrey Joint Centre for Future Materials University of Surrey Guildford Surrey GU2 7XH UK; ^10^ State Key Laboratory of Catalysis Dalian Institute of Chemical Physics Chinese Academy of Sciences Dalian 116023 P. R. China

**Keywords:** electron‐shuttling, heterostructures, hydrogen evolution, interface, Ru‐based electrocatalyst

## Abstract

Ruthenium (Ru) is a promising electrocatalyst for the hydrogen evolution reaction (HER), despite suffering from low activity in non‐acidic conditions due to the high kinetic energy barrier of H_2_O dissociation. Herein, the synthesis of carbon nanosheet‐supported RuP/Ru heterostructures (RuP/Ru@CNS) from a natural polysaccharide is reported and demonstrates its behavior as an effective HER electrocatalyst in non‐acidic conditions. The RuP/Ru@CNS exhibits low overpotential (106 mV at 200 mA·cm^−2^) in alkaline electrolyte, exceeding most reported Ru‐based electrocatalysts. The electron shuttling between Ru atoms at the RuP/Ru interface results in a lowered energy barrier for H_2_O dissociation by electron‐deficient Ru atoms in the pure Ru phase, as well as optimized H^*^ adsorption of electron‐gaining Ru atoms in the neighboring RuP. A low H^*^ spillover energy barrier between Ru atoms at the RuP/Ru interface further boosts HER kinetics. This study demonstrates a sustainable method for the fabrication of efficient Ru‐based electrocatalysts and provides a more detailed understanding of interface effects in HER catalysis.

## Graphical abstract

In the RuP/Ru heterostructure, the electron‐deficient Ru atom in the Ru phase is better able to adsorb and dissociate H_2_O to form H^*^, whereas the electron‐gaining Ru in RuP provides optimized H^*^ adsorption to generate H_2_. The low H^*^ spillover energy barrier between Ru atoms across the RuP/Ru interface facilitates hydrogen transfer from one side of the interface to the other, resulting in efficient non‐acidic HER kinetics.

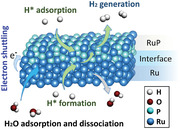

## 1 Introduction

Hydrogen energy draws widespread attention due to its high mass‐energy density and the potential for sustainabile production. Electrochemical water splitting yields high‐purity hydrogen (H_2_) and is widely considered the most promising technology for future large‐scale H_2_ production.^[^ ^1^, ^2^ ^]^ The hydrogen evolution reaction (HER) is heavily dependent on high‐performance electrocatalysts driving efficient and economical water splitting. In non‐acidic conditions, without free protons, the HER process relies on H_2_O dissociation (the Volmer reaction) and the adsorption of H species (H^*^).^[^ ^3^, ^4^ ^]^ Due to the high kinetic energy barrier of H_2_O dissociation (*E*_OH_^*^_‐H_^*^), the HER process usually exhibits slower kinetics in non‐acidic conditions.^[^ ^5^ ^]^ The search for novel electrocatalyst structures with fast HER kinetics is crucial to the development of efficient non‐acidic electrolyzers.

For efficient hydrogen evolution in non‐acidic conditions, electrocatalysts should enable both high H_2_O capture/dissociation and appropriate H^*^ adsorption. Ruthenium (Ru) catalysts have been widely studied as promising candidates for the HER in non‐acidic conditions due to the low *E*_OH_^*^_‐H_^*^ of Ru active sites.^[^ ^6^, ^7^, ^8^ ^]^ Numerous methods have been proposed for enhancing HER activity in Ru‐based electrocatalysts through the rational design of nanostructures. Examples include morphological and size control, alloying, doping, tuning of coordination environments, and the modulation of electronic states at active sites.^[^ ^9^, ^10^, ^11^, ^12^, ^13^, ^14^, ^15^ ^]^ For instance, the Ru‐M (M = Cu, Ni, Mn, etc.) bimetallic nanoalloys can act as efficient HER electrocatalysts at all pH levels. The incorporation of Cu into Ru has been found to accelerate H_2_O capture and activation,^[^ ^10^ ^]^ and Ni doping into Ru was shown to result in electron‐deficient Ru atoms which provide enhanced H^*^ adsorption.^[^ ^16^ ^]^ Heterostructured Ru‐based electrocatalysts provide another route to increased HER activity through careful tuning of the interfacial electronic structure.^[^ ^17^, ^18^, ^19^, ^20^ ^]^ Bi‐phase heterostructures can allow different steps of the HER pathway to occur over different components, thereby improving the overall efficiency of HER catalysis.^[^ ^21^, ^22^ ^]^ Ru metal nanoparticles (NPs) have a high H_2_O dissociation ability.^[^ ^23^ ^]^ When incorporated in a heterostructure with another component providing moderate H^*^ adsorption, a highly efficient HER process occurs at the heterostructure interface. It is known that the introduction of electronegative P atoms into metallic Ru, forming ruthenium phosphides (RuP_x_), can trap charges from Ru atoms and result in a lower hydrogen bonding strength.^[^ ^24^, ^25^ ^]^ This provides motivation for the current work, in which RuP_x_/Ru electrocatalysts were designed to boost HER activity in non‐acidic conditions through optimization of the RuP_x_/Ru interfacial electronic structure for high H_2_O dissociation and optimal H^*^ adsorption. The resulting materials have high density of active sites and were produced via simple synthesis from a sustainable precursor, minimizing cost and maximizing the overall efficiency of electrocatalysis.

Alginate, a complex carbohydrate obtained from seaweed, is an abundant renewable polysaccharide. Its α‐L‐guluronate (G) blocks can spontaneously chelate with polyvalent metal ions (such as Co^2+^, Fe^3+^, Ru^3+^, etc) to form so‐called “egg‐box” structures upon pyrolysis, providing a facile synthesis route for metal‐containing catalytic materials.^[^ ^26^, ^27^, ^28^, ^29^ ^]^ Herein, we synthesized RuP/Ru heterostructures supported on 2D carbon nanosheets (RuP/Ru@CNS) with a high content of Ru species (≈48.3 wt.%) via a facile pyrolysis and phosphatization process using polysaccharide‐alginate as the precursor. The resultant RuP/Ru@CNS electrocatalyst exhibits more efficient HER performance, with low overpotentials of 106 mV at 200 mA·cm^−2^ in 1.0 m KOH and 80 mV at 50 mA·cm^−2^ in 1.0 m PBS electrolytes, than pure RuP@CNS and Ru@CNS, which are themselves superior to the majority of Ru‐based electrocatalysts. Furthermore, electronic structure changes due to interface effects in the RuP/Ru heterostructure have been systematically investigated and correlated with HER behavior. Density functional theory (DFT) calculations demonstrate that a proportion of interfacial electrons transfer from Ru metal to the Ru atoms in RuP, and that this electron shuttling lowers *E*_OH_^*^_‐H_^*^ on Ru atoms in the pure Ru component and optimizes H^*^ adsorption on Ru atoms in the RuP component. Moreover, the low H^*^ spillover energy barrier between Ru atoms at the RuP/Ru interface promotes HER kinetics in non‐acidic conditions.

## 2 Result and Discussion

**Figure 1:**
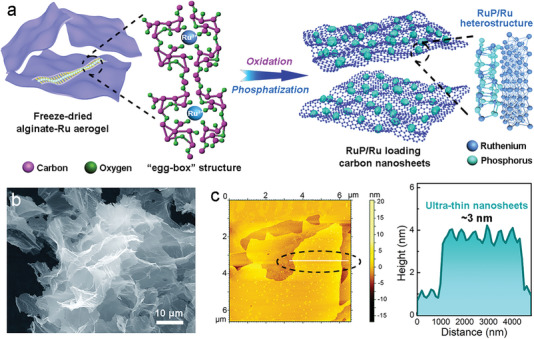
Preparation and morphology of RuP/Ru loaded carbon nanosheets. a) Schematic illustration of RuP/Ru@CNS synthesis. b) FESEM and c) AFM images of RuP/Ru@CNS.

The RuP/Ru@CNS electrocatalyst was synthesized through a facile oxidation and partial phosphatization process using an alginate‐Ru hydrogel precursor (**Figure** **1**a). The alginate‐Ru hydrogel was prepared according to our previously reported method^[^ ^30^ ^]^: Ru^3+^ ions were immobilized and dispersed in alginate‐Ru hydrogels to form an “egg‐box” structure, resulting from coordination with four G‐blocks. After annealing in air, the alginates were converted into a carbon skeleton, while Ru^3+^ species anchored in alginate‐Ru hydrogel precursors were converted to RuO_2_ nanoparticles uniformly immobilized on 2D carbon nanosheets (RuO_2_@CNS, Figure S1, Supporting Information). Finally, ultrathin RuP/Ru@CNS materials were obtained through vapor phosphatization of RuO_2_@CNS at 350 °C for 2, 3, and 4 h. It was found that 3 h RuP/Ru@CNS phosphatization exhibited the best HER performance, and this sample was chosen for further study in this paper. Control samples of single‐component Ru@CNS and RuP@CNS were also prepared (see the Experimental Details Section in Supporting Information). The carbon nanosheet support can enhance electrical conductivity and promote mass diffusion during the HER process.^[^ ^27^, ^30^ ^]^ Field emission scanning electron microscopy (FESEM) images of RuP/Ru@CNS, Ru@CNS, and RuP@CNS show a typical 2D nanosheet morphology (Figure 1b; Figure S2, Supporting Information). This morphology has a high specific surface area, promoting mass transfer to improve HER kinetics, and the high dispersion of RuP/Ru on the CNS surface ensures that RuP/Ru sites are fully utilized during the HER. Atomic force microscopy (AFM) images and corresponding height profiles show that the RuP/Ru@CNS, Ru@CNS, and RuP@CNS samples are ≈3, ≈3, and ≈7 nm thick, respectively (Figure 1c; Figure S3, Supporting Information).

**Figure 2:**
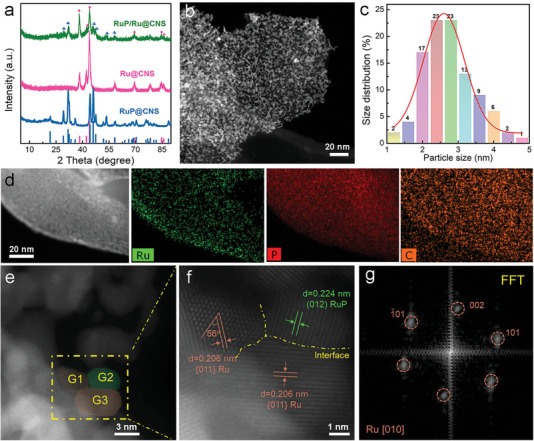
Structural characterization of the prepared electrocatalysts. a) XRD patterns of RuP/Ru@CNS, Ru@CNS and RuP@CNS. b) HAADF‐STEM image and c) diameter distribution of RuP/Ru nanoparticles in RuP/Ru@CNS. d) HAADF‐STEM image and corresponding EDS elemental maps of Ru, P, and C in RuP/Ru@CNS. e,f) High‐resolution HAADF‐STEM images of RuP/Ru@CNS and g) corresponding FFT of nanoparticle G1 in (e).

Crystal structures of the samples were confirmed by X‐ray diffraction (XRD). The XRD pattern of the RuO_2_@CNS matches well with RuO_2_ (JCPDS no.008–4618, Figure S1, Supporting Information). After phosphating RuO_2_@CNS at 350 °C, the XRD pattern of RuP/Ru@CNS contains diffraction peaks of both Ru and RuP. As shown in **Figure** **2**a, the diffraction peaks at 21.7°, 29.2°, 31.8°, 44.2°, 46.1°, 47.5°, 53.4°, and 58.3° are consistent with the (101), (002), (011), (202), (211), (103), (013), and (020) planes of RuP (JCPDS no. 65−1863), while the peaks located at 38.4°, 42.2°, 44.0°, 58.3°, 69.4°, 78.4°, 84.7°, and 85.9° can be indexed to the (100), (002), (101), (102), (110), (103), (112), and (201) planes of Ru (JCPDS no. 89−4903), suggesting successful synthesis of RuP/Ru heterostructures. The content of Ru species in Ru@CNS, RuP@CNS, and RuP/Ru@CNS is 51.2, 44.5, and 48.3 wt.%, respectively, as measured by inductively coupled plasma mass spectrometry (ICP‐MS). High‐angle annular dark‐field scanning transmission electron microscopy (HAADF‐STEM) images further confirm that all of the samples have a typical 2D nanosheet structure (Figure S4, Supporting Information) with nanoparticles evenly distributed on the carbon nanosheet support. RuP/Ru@CNS crystals are agglomerated together, and the irregularly shaped Ru and/or RuP NPs have an average crystallite size of ≈2–4 nm (Figure 2b,c). Although the phosphating process can affect the size and dispersion of Ru and/or RuP NPs (Figure 2b; Figure S5, Supporting Information), the small NP crystallite size ensures a good degree of dispersion and active site accessibility, providing superior catalytic activity.^[^ ^31^ ^]^

Energy dispersive X‐ray spectroscopy (EDS) STEM elemental maps of RuP/Ru@CNS reveal a uniform distribution of Ru, P, O, and C within the nanosheets (Figure 2d). To confirm the local crystal structure, atomic‐resolution HAADF‐STEM images were acquired from an area containing several NPs (labeled G1, G2, and G3 in Figure 2e,f). The interfaces between different particles are marked by dashed yellow lines. As can be seen, the particles aggregated together with different crystal orientations. The fast fourier transform (FFT) pattern in Figure 2g demonstrates the 2D lattice structure of G1 and can be indexed to the Ru crystal structure viewed along the [010] zone axis. For grains G2 and G3, only lattice planes are observed; the interplanar spacing can be assigned to RuP for G2 (0.224 nm lattice spacing correspond to the (012) planes of RuP) and Ru for G3 (0.206 nm lattice spacing corresponds to the {011} planes of Ru). Based on the observed morphology, a large number of Ru/RuP interfaces exist in the RuP/Ru@CNS sample. High‐resolution images of Ru@CNS and RuP@CNS show lattice planes corresponding to Ru or RuP respectively (Figure S5, Supporting Information), supporting the XRD conclusion that these contain only a single Ru phase.

**Figure 3:**
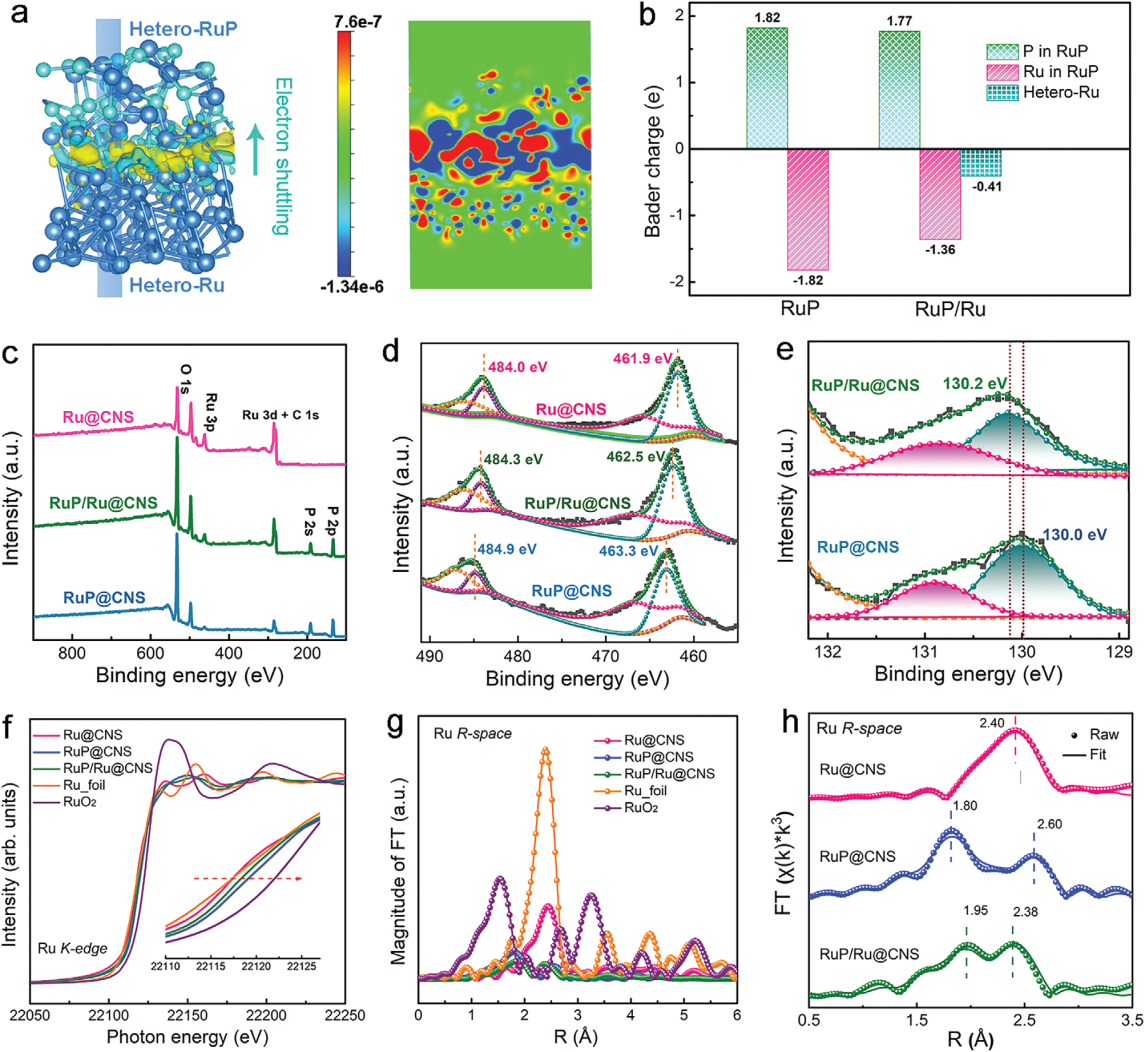
Electronic and structural characterization of the prepared electrocatalysts. a) The calculated charge density of the RuP/Ru interface. b) Bader charge analysis diagram of RuP and the RuP/Ru heterostructure. c) XPS full survey spectra and d) high‐resolution XPS spectra of Ru 3p for Ru@CNS, RuP/Ru@CNS, and RuP@CNS. e) High‐resolution P 2p XPS spectra of RuP/Ru@CNS and RuP@CNS. Ru K‐edge f) XANES and g) the FT‐EXAFS and h) best‐fit EXAFS spectra in R space for Ru@CNS, RuP@CNS, and RuP/Ru@CNS.

The electronic state of Ru active sites at RuP/Ru interfaces was investigated with DFT. Models of pure Ru, pure RuP, and RuP/Ru were constructed for the calculations (Figure S6, Supporting Information). In the heterostructured RuP/Ru model, there is an electron shuttling phenomenon observed at the RuP/Ru interface between Ru atoms in the RuP component (Hetero‐RuP) and those in the Ru component (Hetero‐Ru), where part of the charge of the Hetero‐Ru atom is transferred to Hetero‐RuP (**Figure** **3**a; Figure S7, Supporting Information). Bader charge analysis further demonstrates a propensity for electron transfer in RuP/Ru. This is not seen in pure Ru or RuP. The charge number of Ru atoms in pure RuP (−1.82 eV) is more negative than that of Hetero‐RuP at the heterostructure interface (−1.36 eV), whereas the charge number of Hetero‐Ru atoms at the interface is −0.41 eV, revealing that charge from Hetero‐Ru atoms is readily transferred to Ru atoms in Hetero‐RuP at the RuP/Ru interface (Figure 3b). The chemical states of Ru and P were further analyzed by X‐ray photoelectron spectroscopy (XPS). XPS survey spectra reveal that, as expected, Ru is present in all samples, while P is present in RuP/Ru@CNS and RuP@CNS but not in Ru@CNS (Figure 3c). In high‐resolution XPS spectra of Ru 3p, the two peaks at binding energies of around 462 and 484 eV can be associated with Ru 3p_3/2_ and Ru 3p_1/2_ respectively (Figure 3d). The Ru 3p_3/2_ and Ru 3p_1/2_ peaks of RuP@CNS are located at 462.5 and 484.3 eV, corresponding to the expected binding of Ru─P bonds.^[^ ^28^, ^29^ ^]^ For Ru@CNS and RuP@CNS, the Ru 3p peaks of RuP/Ru@CNS are positively and negatively shifted, respectively, indicating the Ru atoms in RuP/Ru@CNS are electron‐deficient relative to Ru@CNS and electron‐rich relative to RuP@CNS.^[^ ^32^, ^33^ ^]^ The peaks at ≈134.5 and ≈130.0 eV in the P 2p XPS spectra of RuP/Ru@CNS and RuP@CNS can be assigned to P‐O and Ru─P bonds (Figure 3e). In RuP/Ru@CNS the P 2p_3/2_ peak at 130.2 eV shows a positive shift compared to that of RuP@CNS (at 130.0 eV), illustrating electron‐deficient P atoms in Ru─P bonds at RuP/Ru heterostructures.

The chemical environment of the RuP/Ru heterostructure was further studied with synchrotron‐based X‐ray absorption spectroscopy. X‐ray absorption near‐edge structure (XANES) analysis of RuP/Ru@CNS, Ru@CNS, and RuP@CNS is compared to control samples of Ru foil and RuO_2_ in Figure 3f. XANES shows that the Ru K‐edge absorption energies in RuP/Ru@CNS are higher than those of Ru@CNS and Ru foil, but lower than those of RuP@CNS, suggesting that the average valence state of Ru atoms in RuP/Ru is between Ru^3+^ and metallic Ru.^[^ ^34^, ^35^ ^]^ Fourier‐transform‐extended X‐ray absorption fine structure (FT‐EXAFS) spectra and their fitting results further clarified the above structural transformation and provided accurate structural information for transformed species (Figure 3g,h; Figure S8, Supporting Information). The FT‐EXAFS spectrum of Ru@CNS shows a main peak at 2.40 Å, similar to Ru foil and attributable to metallic Ru–Ru bonds in Ru nanoparticles. The FT‐EXAFS spectrum of RuP@CNS exhibits a primary peak at around 1.80 Å, assigned to Ru─P bonds in RuP. A decreased Ru‐Ru bond distance (2.38 Å) and increased Ru─P bond distance (1.95 Å) in RuP/Ru@CNS compared with those of Ru@CNS and RuP@CNS may be due to the interaction between Ru and RuP phases in the heterostructure.^[^ ^36^, ^37^ ^]^ In combination, these XPS and XANES results demonstrate electron shuttling at the RuP/Ru interface and the formation of positively charged Ru species in RuP/Ru@CNS, matching well with our theoretical calculations.

**Figure 4:**
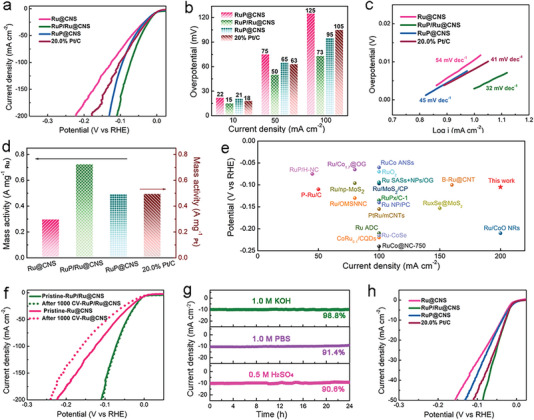
Electrocatalytic HER performance of the prepared electrocatalysts. a) *IR*‐corrected HER LSV curves, b) overpotential values of Ru@CNS, RuP/Ru@CNS RuP@CNS and 20% Pt/C at j = 10, 50 and 100 mA·cm^−2^, respectively, in 1.0 m KOH electrolyte, c) Tafel plots and d) mass activity of RuP/Ru@CNS, RuP/Ru@CNS, RuP@CNS and 20.0% Pt/C in 1.0 m KOH. e) Comparison of overpotentials at 10 mA·cm^−2^ and the Tafel slope of RuP/Ru@CNS with previously reported Ru‐based electrocatalysts in alkaline media. f) Cycling stability of RuP/Ru@CNS and Ru@CNS in 1.0 m KOH electrolyte. g) The time dependence of current density curves at static overpotentials at 10 mA·cm^−2^ for 24 h in 1.0 m KOH, 1.0 m PBS, and 0.5 m H_2_SO_4_ electrolytes. h) *IR*‐corrected HER LSV curves for Ru@CNS, RuP/Ru@CNS, RuP@CNS and 20% Pt/C in a 1.0 m PBS electrolyte.

The HER performances of Ru@CNS, RuP/Ru@CNS, and RuP@CNS catalysts (each with 0.5 mg·cm^−2^ electrode loading) were first examined in a 1.0 m KOH electrolyte. 20.0% Pt/C (1.0 mg·cm^−2^ electrode loading) was used as a standard reference sample. *IR*‐corrected linear sweep voltammetry (LSV) curves in a 1.0 m KOH electrolyte show that the HER performance of Ru@CNS is inferior to those of RuP@CNS and RuP/Ru@CNS, implying that RuP has significantly improved HER activity compared to Ru metal (**Figure** **4**a). Notably, the RuP/Ru@CNS only requires overpotentials of 15, 50, and 73 mV at 10, 50, and 100 mA·cm^−2^ respectively, values which are much smaller than the corresponding overpotentials of Ru@CNS, RuP@CNS, and the 20.0% Pt/C reference material (Figure 4b). The reaction kinetics of the samples were evaluated using Tafel slope values derived from LSV curves. RuP/Ru@CNS achieves the lowest Tafel slope value of 32 mV·dec^−1^, while Ru@CNS has the highest (54 mV·dec^−1^), followed by RuP@CNS (45 mV·dec^−1^) and then 20.0% Pt/C (41 mV·dec^−1^), confirming the excellent catalytic activity of RuP/Ru@CNS and showing that the Volmer–Tafel pathway is the rate‐limiting step (Figure 4c). RuP/Ru@CNS also shows a higher noble metal mass activity of 0.724 A mg^−1^_Ru_ than Ru@CNS, RuP@CNS, and 20.0 wt.% Pt/C (Figure 4d). The number of exposed active sites of an electrocatalyst can be estimated by measuring the double‐layer capacitance (*C*_dl_). The RuP/Ru@CNS catalyst presents a *C*_dl_ value of 47.20 mF·cm^−2^ in 1.0 m KOH, superior to those of the Ru@CNS and RuP@CNS catalysts (Figures S9 and S10, Supporting Information). The excellent HER activity of RuP/Ru@CNS can be attributed to interface effects between RuP and Ru, enabling the material to significantly outperform most previously reported Ru‐based HER electrocatalysts in alkaline electrolytes (Figure 4e).^[^ ^9^, ^25^, ^38^, ^39^, ^40^, ^41^, ^42^, ^43^, ^44^, ^45^, ^46^, ^47^, ^48^, ^49^, ^50^, ^51^, ^52^, ^53^ ^]^

To evaluate the HER durability of the RuP/Ru@CNS catalyst, cyclic voltammogram (CV) scanning, and chronoamperometry tests were performed in 1.0 m KOH, 1.0 m PBS, and 0.5 m H_2_SO_4_ electrolytes. Significant differences were observed in the LSV curves for the Ru@CNS catalyst before and after 1000 cycles, revealing the material's relatively poor durability (Figure 4f; Figure S11, Supporting Information). In contrast, the LSV curves of the RuP/Ru@CNS catalyst show negligible degradation. Similar behavior is observed in the corresponding time‐dependent potential curves (Figure 4g; Figure S12, Supporting Information). In order to evaluate the performance of the catalyst in practical applications, we assembled an anion‐exchange membrane (AEM) electrolyzer with commercial RuO_2_ and RuP/Ru@CNS as the anode and cathode respectively, which demonstrated excellent performance. Only 1.975 V was required at a current density of 1200 mA cm^−2^ (Figure S19, Supporting Information), which is significantly better than the combination of commercial RuO_2_ and commercial 20.0 wt. Pt/C (2.577 V). The high HER stability of RuP/Ru@CNS can be ascribed to the electronic and structural stability of the RuP/Ru heterostructure, as evidenced by structural characterization of the RuP/Ru@CNS catalyst after 24 h of galvanostatic testing at 10 mA cm^−2^. The Ru 3p and P 2p peaks of RuP/Ru@CNS had hardly any shift after the stability test, and it retained a similar nanostructure to that observed in the pristine catalyst (Figures S13 and S14, Supporting Information). The HER performance of RuP/Ru@CNS was further probed in 1.0 m PBS and 0.5 m H_2_SO_4_ electrolytes. Remarkably, RuP/Ru@CNS also delivers impressive HER activities in neutral and acidic conditions (Figure 4h; Figure S15, Supporting Information), with overpotential values lower than those of Ru@CNS and RuP@CNS (Figure S16, Supporting Information). The corresponding Tafel slopes of RuP/Ru@CNS in 1.0 m PBS and 0.5 m H_2_SO_4_ electrolytes are as low as 39 and 28 mV·dec^−1^ respectively (Figure S17, Supporting Information). The 2D structure of RuP/Ru@CNS and its highly exposed active sites promote charge transfer kinetics. Electrochemical impedance spectroscopy (EIS) at an overpotential of 100 mV was performed to investigate the HER charge transfer kinetics of the catalysts. RuP/Ru@CNS has lower charge transfer resistance (*R*_ct_) than Ru@CNS and RuP@CNS in 1.0 m KOH, 1.0 m PBS, and 0.5 m H_2_SO_4_ electrolytes (Figure S18, Supporting Information), indicating that the formation of a RuP/Ru heterostructure can accelerate interfacial charge transfer kinetics.

**Figure 5:**
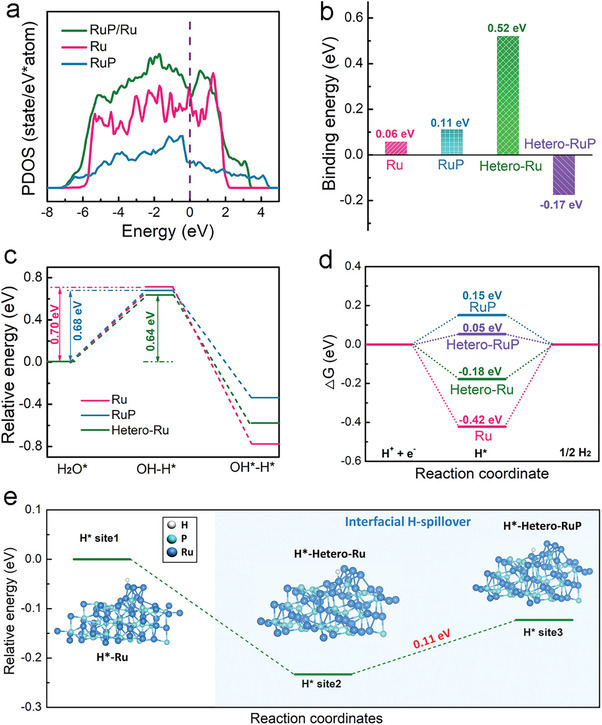
Theoretical calculations and analysis of the HER mechanism on the prepared electrocatalysts. a) PDOS of the Ru, RuP, and RuP/Ru models. b) The binding energy of H_2_O with Ru atoms on Ru and RuP surfaces and at the RuP/Ru interface. c) The calculated kinetic barrier of H_2_O dissociation on Ru atoms in Ru, RuP, and Hetero‐Ru. d) The calculated hydrogen adsorption free energy diagram for Ru atoms in Ru, RuP/Ru, and RuP. e) The calculated hydrogen spillover energy barrier between Ru atoms at the RuP/Ru interface.

To better understand the exceptional non‐acidic HER activity of the prepared RuP/Ru heterostructure, DFT was used to calculate the energy barrier to HER. The partial density of states (PDOS) calculated for the three structural models reveal that the density of states across the Fermi level in RuP/Ru is similar to that of pure Ru and higher than that of RuP, suggesting higher conductivity in the RuP/Ru heterostructure compared to RuP (**Figure** **5**a). The HER pathway under alkaline and neutral conditions begins with H_2_O dissociation to form a H^*^/OH^*^ intermediate, followed by H^*^ adsorption and finally production of H_2_. The process as a whole is limited by *E*_OH_^*^_‐H_^*^ or the Gibbs free energy of H^*^ adsorption (*ΔG*_H_^*^). The binding energies of H_2_O with Ru atoms in Ru, RuP, Hetero‐Ru, and Hetero‐RuP are found to be 0.06, 0.11, 0.52, and −0.17 eV, respectively (Figure 5b), demonstrating that Hetero‐RuP is more favorable for H_2_O adsorption, needed to drive H_2_O dissociation, than either Ru or RuP.

Figure 5c,d presents *E*_OH_^*^_‐H_^*^ and *ΔG*_H_^*^ values for Ru atoms at the RuP/Ru interface in pure Ru and in pure RuP. The *E*_OH_^*^_‐H_^*^ values of Ru atoms in pure Ru and pure RuP are 0.70 and 0.68 eV, respectively. In contrast, the energy barrier for H_2_O dissociation in Hetero‐Ru reduces to 0.64 eV, indicating that slightly electron‐deficient Ru atoms can efficiently dissociate H_2_O and facilitate the HER in non‐acidic conditions. The *ΔG*_H_^*^ values of Ru and Hetero‐Ru are −0.42 and −0.18 eV respectively, implying that the H^*^ intermediate can spontaneously adsorb on these Ru atoms, but that it is difficult to desorb the resulting H_2_. For Ru atoms in Hetero‐RuP, the *ΔG*_H_^*^ value significantly increases to 0.05 eV, which is more favorable for H_2_ desorption (Figure 5d). Furthermore, the small difference in work function between Ru and RuP (*ΔΦ* = 0.07 eV; Figure S20, Supporting Information) may trigger hydrogen spillover at the RuP/Ru interface.^[^ ^54^, ^55^, ^56^ ^]^ As illustrated in Figure 5e, the H^*^ spillover energy barrier from a Ru atom in Hetero‐Ru (site 2) to a Ru atom in Hetero‐RuP (site 3) is only 0.11 eV, so H^*^ generated on Hetero‐Ru can easily transfer to Hetero‐RuP and desorb to produce H_2_. In the RuP/Ru heterostructure, Hetero‐Ru is modified by the proximity of electronegative P atoms in Hetero‐RuP, with partial charge shuttling from Ru atoms in Hetero‐Ru to Ru atoms in Hetero‐RuP. Electron‐deficient Ru atoms in Hetero‐Ru are more able to adsorb and dissociate H_2_O to form H^*^, and the electron‐gaining Ru in Hetero‐RuP favors H^*^ adsorption to generate H_2_. The low H^*^ spillover energy barrier between Ru atoms across the RuP/Ru interface thereby improves HER kinetics in non‐acidic conditions.

## 3 Conclusion

In summary, a heterostructured RuP/Ru@CNS electrocatalyst was synthesized using a sustainable polysaccharide‐alginate as the carbon precursor through a facile pyrolysis and partial phosphatization process. Compared with pure Ru‐ and RuP‐containing samples (Ru@CNS and RuP@CNS), heterostructured RuP/Ru@CNS shows significantly enhanced non‐acidic HER performance, with a low overpotential of 106 mV at 200 mA·cm^−2^ in an alkaline electrolyte. This excellent HER performance is ascribed to interface effects at the RuP/Ru boundary. Theoretical calculations and chemical analysis reveal that electron shuttling across the RuP/Ru interface results in charge redistribution between Ru atoms, reducing the energy barrier for H_2_O dissociation on the Ru component and increasing the interaction between H^*^ and the RuP component. A low H^*^ spillover energy barrier between Ru atoms across the RuP/Ru interface additionally promotes non‐acidic HER kinetics. This study demonstrates a feasible strategy for designing efficient Ru‐based HER electrocatalysts and provides an improved understanding of HER activity enhancement through the tuning of interfacial effects in heterostructured catalysts. It is hoped that these insights can be extended to other transition metal electrocatalysts.

## Conflict of Interest

The authors declare no conflict of interest.

## Supporting information

Supporting Information

## Data Availability

The data that support the findings of this study are available from the corresponding author upon reasonable request.

## References

[1] M. Dresselhaus , I. Thomas , Nature 2001, 414, 332. 11713539 10.1038/35104599

[2] S. Chu , A. Majumdar , Nature 2012, 488, 294. 22895334 10.1038/nature11475

[3] S. Anantharaj , S. Noda , V. Jothi , S. Yi , M. Driess , P. W. Menezes , Angew. Chem., Int. Ed. 2021, 60, 18981. 10.1002/anie.202015738 PMC8451938 33411383

[4] Y. Zheng , Y. Jiao , A. Vasileff , S. Z. Qiao , Angew. Chem. Int. Ed. 2018, 57, 7568. 10.1002/anie.201710556 29194903

[5] Y. Jiao , Y. Zheng , M. Jaroniec , S. Z. Qiao , Chem. Soc. Rev. 2015, 44, 2060. 25672249 10.1039/c4cs00470a

[6] S. Zhang , J. Li , E. Wang , ChemElectroChem 2020, 7, 4526.

[7] S. Bae , J. Mahmood , I. Jeon , J.‐B. Baek , Nanoscale Horiz. 2020, 5, 43.

[8] J. Cai , R. Javed , D. Ye , H. Zhao , J. Zhang , Mater. Chem. A. 2020, 8, 22467.

[9] Y. Wu , X. Li , Y. Wei , Z. Fu , W. Wei , X. Wu , Q. Zhu , Q. Xu , Adv. Mater. 2021, 33, 2006965. 10.1002/adma.202006965 33598974

[10] Q. Wu , M. Luo , J. Han , W. Peng , Y. Zhao , D. Chen , M. Peng , J. Liu , F. Groot , Y. Tan , ACS Energy Lett. 2020, 5, 192.

[11] J. Su , Y. Yang , G. Xia , J. Chen , P. Jiang , Q. Chen , Nat. Commun. 2017, 8, 14969. 28440269 10.1038/ncomms14969 PMC5413983

[12] J. Wang , Z. Wei , S. Mao , H. Li , Y. Wang , Energy Environ. Sci. 2018, 11, 800.

[13] X. Liu , F. Liu , J. Yu , G. Xiong , L. Zhao , Y. Sang , S. Zuo , J. Zhang , H. Liu , W. Zhou , Adv. Sci. 2020, 7, 2001526. 10.1002/advs.202001526 PMC7507474 32995134

[14] J. Yang , B. Chen , X. Liu , W. Liu , Z. Li , J. Dong , W. Chen , W. Yan , T. Yao , X. Duan , Y. Wu , Y. Li , Angew. Chem. 2018, 130, 9639. 10.1002/anie.201804854 29897158

[15] P. Zhu , X. Xiong , D. Wang , Nano. Res. 2022, 15, 5792

[16] Y. Liu , X. Li , Q. Zhang , W. Li , Y. Xie , H. Liu , L. Shang , Z. Liu , Z. Chen , L. Gu , Z. Tang , T. Zhang , S. Lu , Angew. Chem., Int. Ed. 2020, 59, 1718. 10.1002/anie.201913910 31799763

[17] J. Mao , C. He , J. Pei , W. Chen , D. He , Y. He , Z. Zhuang , C. Chen , Q. Peng , D. Wang , Y. Li , Nat. Commun. 2018, 9, 4958. 30470747 10.1038/s41467-018-07288-6 PMC6251903

[18] Y. Liu , S. Liu , Y. Wang , Q. Zhang , L. Gu , S. Zhao , D. Xu , Y. Li , J. Bao , Z. Dai , J. Am. Chem. Soc. 2018, 140, 2731. 29415541 10.1021/jacs.7b12615

[19] X. Xiao , X. Wang , X. Jiang , S. Song , D. Huang , L. Yu , Y. Zhang , S. Chen , M. Wang , Y. Shen , Z. Ren , Small Methods 2020, 4, 1900796.

[20] Y. Hu , T. Chao , Y. Li , P. Liu , T. Zhao , G. Yu , C. Chen , X. Liang , H. Jin , S. Niu , W. Chen , D. Wang , Y. Li , Angew. Chem., Int. Ed. 2023, 62, e202308800. 10.1002/anie.202308800 37428114

[21] J. Zhu , Y. Guo , F. Liu , H. Xu , L. Gong , W. Shi , D. Chen , P. Wang , Y. Yang , C. Zhang , J. Wu , J. Luo , S. Mu , Angew. Chem., Int. Ed. 2021, 60, 12328. 10.1002/anie.202101539 33634585

[22] J. Xu , T. Liu , J. Li , B. Li , Y. Liu , B. Zhang , D. Xiong , I. Amorim , W. Li , L. Liu , Energy Environ. Sci. 2018, 11, 1819.

[23] L. Zhang , H. Jang , Y. Wang , Z. Li , W. Zhang , M. Kim , D. Yang , S. Liu , X. Liu , J. Cho , Adv. Sci. 2021, 8, 2004516. 10.1002/advs.202004516 PMC8336516 34085783

[24] J. Zhu , S. Li , M. Xiao , X. Zhao , G. Li , Z. Bai , M. Li , Y. Hu , R. Feng , W. Liu , R. Gao , D. Su , A. Yu , Z. Chen , Nano Energy 2020, 77, 105212.

[25] Y. Zhao , X. Wang , G. Cheng , W. Luo , ACS Catal. 2020, 10, 11751.

[26] L. Zhang , T. Liu , N. Chen , Y. Jia , R. Cai , W. Theis , X. Yang , Y. Xia , D. Yang , X. Yao , J. Mater. Chem. A. 2018, 6, 18417.

[27] H. Li , X. Zhao , H. Liu , S. Chen , X. Yang , C. Lv , H. Zhang , X. She , D. Yang , Small 2018, 14, 1802824. 10.1002/smll.201802824 30350551

[28] L. Zhang , H. Jang , H. Liu , M. Kim , D. Yang , S. Liu , X. Liu , J. Cho , Angew. Chem., Int. Ed. 2021, 60, 18821. 10.1002/anie.202106631 34121280

[29] C. Lv , Y. Zhu , W. Zhang , W. Xu , J. Ren , H. Liu , X. Yang , R. Cai , S. Jin , D. Li , D. Yang , Mater. Today Energy 2021, 21, 100834.

[30] D. Zheng , C. Lv , X. Zhang , S. Chen , H. Liu , Y. Sun , X. She , D. Li , D. Yang , Mater. Today Sustain. 2021, 13, 100074.

[31] H. Kang , L. Zhu , S. Li , S. Yu , Y. Niu , B. Zhang , W. Chu , X. Liu , S. Perathoner , G. Centi , Y. Liu , Nat. Catal. 2023, 6, 1062.

[32] R. Ge , S. Wang , J. Su , Y. Dong , Y. Lin , Q. Zhang , L. Chen , Nanoscale 2018, 10, 13930. 30019735 10.1039/c8nr03554g

[33] W. Li , Y. Zhao , Y. Liu , M. Sun , Angew. Chem., Int. Ed. 2021, 60, 3290. 10.1002/anie.202013985 33105050

[34] X. Jiang , H. Jang , S. Liu , Z. Li , M. G. Kim , C. Li , Q. Qin , X. Liu , J. Cho , Angew. Chem., Int. Ed. 2021, 60, 4110. 10.1002/anie.202014411 33174362

[35] K. Jiang , M. Luo , Z. Liu , M. Peng , D. Chen , Y. Lu , T. Chan , F. Groot , Y. Tan , Nat. Commun. 2021, 12, 1687. 33727537 10.1038/s41467-021-21956-0 PMC7966786

[36] P. Su , W. Pei , X. Wang , Y. Ma , Q. Jiang , J. Liang , S. Zhou , J. Zhao , J. Liu , G. Q. Lu , Angew. Chem., Int. Ed. 2021, 60, 16044. 10.1002/anie.202103557 33960092

[37] J. Shan , C. Guo , Y. Zhu , S. Chen , L. Song , Chem 2019, 5, 445.

[38] X. Sun , W. Li , J. Chen , X. Yang , B. Wu , Z. Wang , B. Li , H. Zhang , J. Colloid Interf. Sci. 2022, 616, 338. 10.1016/j.jcis.2022.02.072 35219199

[39] W. Wu , Y. Wu , D. Zheng , K. Wang , Z. Tang , Electrochim. Acta 2019, 320, 134568.

[40] J.‐X. Guo , D.‐Y. Yan , K.‐W. Qiu , C. Mu , D. Jiao , J. Mao , H. Wang , T. Ling , J. Energy Chem. 2019, 37, 143.

[41] W. Luo , Y. Wang , X. Li , C. Cheng , Nanotechnology 2020, 31, 295401. 32203950 10.1088/1361-6528/ab824b

[42] L. Guo , F. Luo , F. Guo , Q. Zhang , K. Qu , Z. Yang , W. Cai , Chem. Commun. 2019, 55, 7623. 10.1039/c9cc03675j 31194204

[43] J. Liu , Y. Zheng , D. Zhu , A. Vasileff , T. Ling , S.‐Z. Qiao , Nanoscale 2017, 9, 16616. 29075731 10.1039/c7nr06111k

[44] W. Li , Y. Zhao , Y. Liu , M. Sun , G. I. N. Waterhouse , B. Huang , K. Zhang , T. Zhang , S. Lu , Angew. Chem., Int. Ed. 2021, 60, 3290. 10.1002/anie.202013985 33105050

[45] M. Wang , Z. Dang , M. Prato , U. Petralanda , I. Infante , D. V. Shinde , L. De Trizio , L. Manna , ACS Appl. Nano Mater. 2019, 2, 5695.

[46] M. Ming , Y. Zhang , C. He , L. Zhao , S. Niu , G. Fan , J. Hu , Small 2019, 15, 1903057. 10.1002/smll.201903057 31701640

[47] K. Jiang , M. Luo , Z. Liu , M. Peng , D. Chen , Y.‐R. Lu , T.‐S. Chan , F. M. F. de Groot , Y. Tan , Nat. Commun. 2021, 12, 1687. 33727537 10.1038/s41467-021-21956-0 PMC7966786

[48] D. Cao , J. Wang , H. Xu , D. Cheng , Small 2021, 17, 2101163. 10.1002/smll.202101163 34213837

[49] Y. Dang , T. Wu , H. Tan , J. Wang , C. Cui , P. Kerns , W. Zhao , L. Posada , L. Wen , S. L. Suib , Energy Environ. Sci. 2021, 14, 5433.

[50] P. Su , W. Pei , X. Wang , Y. Ma , Q. Jiang , J. Liang , S. Zhou , J. Zhao , J. Liu , G. Q. (Max) Lu , Angew. Chem., Int. Ed. 2021, 60, 16044. 10.1002/anie.202103557 33960092

[51] Z. Chen , W. Chen , L. Zheng , T. Huang , J. Hu , Y. Lei , Q. Yuan , X. Ren , Y. Li , L. Zhang , S. Huang , S. Ye , Q. Zhang , X. Ouyang , X. Sun , J. Liu , Sci. China Chem. 2022, 65, 521.

[52] B. Pang , X. Liu , T. Liu , T. Chen , X. Shen , W. Zhang , S. Wang , T. Liu , D. Liu , T. Ding , Z. Liao , Y. Li , C. Liang , T. Yao , Energy Environ. Sci. 2022, 15, 102.

[53] C. Cai , K. Liu , Y. Zhu , P. Li , Q. Wang , B. Liu , S. Chen , H. Li , L. Zhu , H. Li , J. Fu , Y. Chen , E. Pensa , J. Hu , Y. Lu , T. Chan , E. Cortés , M. Liu , Angew. Chem., Int. Ed. 2022, 61, e202113664. 10.1002/anie.202113664 PMC9300137 34822728

[54] J. Li , J. Hu , M. Zhang , W. Gou , S. Zhang , Z. Chen , Y. Qu , Y. Ma , Nat. Commun. 2021, 12, 3502. 34108475 10.1038/s41467-021-23750-4 PMC8190308

[55] M. Xiong , Z. Gao , Y. Qin , ACS Catal. 2021, 11, 3159.

[56] J. Wei , S. Qin , J. Liu , X. Ruan , Z. Guan , H. Yan , D. Wei , H. Zhang , J. Cheng , H. Xu , Z. Tian , J. Li , Angew. Chem., Int. Ed. 2020, 59, 10343. 10.1002/anie.202000426 32207867

