# Supplementary material for: A Sustainable Route to Ruthenium Phosphide (RuP)/Ru Heterostructures with Electron‐Shuttling of Interfacial Ru for Efficient Hydrogen Evolution
Source: Adv Sci (Weinh). 2024 Mar 28;11(22):2309869. doi: 10.1002/advs.202309869 (PMC11165549; doi:10.1002/advs.202309869)
Supplement: Supplementary file 1 — Supporting Information [file ADVS-11-2309869-s001.pdf]

ADVANCED SCIENCE
Open Access

Supporting Information

for *Adv. Sci.*, DOI 10.1002/advs.202309869

A Sustainable Route to Ruthenium Phosphide (RuP)/Ru Heterostructures with Electron-Shuttling of Interfacial Ru for Efficient Hydrogen Evolution

Daohao Li, Rongsheng Cai, Dongyong Zheng, Jun Ren, Chung-Li Dong, Yu-Cheng Huang, Sarah J. Haigh, Xien Liu, Feilong Gong, Yiming Liu, Jian Liu* and Dongjiang Yang**

Supporting Information

A Sustainable Route to Ruthenium Phosphide (RuP)/Ru Heterostructures with Electron-Shuttling of Interfacial Ru for Efficient Hydrogen Evolution

Daohao Li,[#] *Rongsheng Cai*,[#] *Dongyong Zheng*, *Jun Ren*, *Chung-Li Dong*, *Yu-Cheng Huang*, *Sarah J. Haigh*, *Xien Liu*, *Feilong Gong*, *Yiming Liu*,* *Jian Liu*,* and *Dongjiang Yang**

Abstract: Ruthenium (Ru) is a promising electrocatalyst for the hydrogen evolution reaction (HER), despite suffering from low activity in non-acidic conditions due to the high kinetic energy barrier of H_2O dissociation. Herein, we report the synthesis of carbon nanosheet-supported RuP/Ru heterostructures (RuP/Ru@CNS) from a natural polysaccharide and demonstrate its behavior as an effective HER electrocatalyst in non-acidic conditions. The RuP/Ru@CNS exhibits low overpotential (106 mV at 200 $mA \cdot cm^{-2}$) in alkaline electrolyte, exceeding most reported Ru-based electrocatalysts. The electron shuttling between Ru atoms at the RuP/Ru interface results in a lowered energy barrier for H_2O dissociation by electron-deficient Ru atoms in the pure Ru phase, as well as optimized H* adsorption of electron-gaining Ru atoms in the neighboring RuP. A low H* spillover energy barrier between Ru atoms at the RuP/Ru interface further boosts HER kinetics. This study demonstrates a sustainable method for the fabrication of efficient Ru-based electrocatalysts and provides a more detailed understanding of interface effects in HER catalysis.

Experimental and Procedures

Materials.

Ruthenium(III) chloride hydrate ($RuCl_3 \cdot xH_2O$; 35.0~42.0% Ru basis), sodium alginate (SA) and sodium hypophosphite monohydrate ($NaPO_2H_2 \cdot H_2O$; 99%) were purchased from Aladdin. Nafion 117 solution (~5%) was purchased from Sigma-Aldrich. All reagents were used without further purification.

Synthesis of alginate-Ru aerogels.

In a typical procedure, 1.0 g SA was dissolved in 100 mL distilled water and fast-stirred at room temperature for 3 hours to form a uniform 1 wt% SA solution. The SA solution was then added to Ru^{3+} ions in aqueous solution with a syringe needle and slow-stirred to form crosslinked alginate-Ru hydrogels at room temperature. After 2 hours, the alginate-Ru hydrogels were filtered from the mixed aqueous solution and washed to remove the free metal ions from the surface with ultra-pure water. The hydrogels were finally frozen with liquid nitrogen and dehydrated via a freeze-drying process under −80 °C for 24 h to obtain alginate-Ru aerogels.

Synthesis of RuP/Ru@CNS.

RuP/Ru@CNS samples were prepared by an oxidation and vapor phosphatization process in a tube furnace. Alginate-Ru aerogels were annealed at 300°C for 2 h at a ramp rate of 5 $°C \cdot min^{-1}$ in air. After cooling to room temperature, the products were collected. To prepare RuP/Ru@CNS, 50 mg of the hydrogel precursor and 500 mg $NaPO_2H_2 \cdot H_2O$ were placed in two porcelain boats, with $NaPO_2H_2 \cdot H_2O$ at the upstream side of the tube furnace. The dosage of $NaPO_2H_2 \cdot H_2O$ is sufficiently excessive for phosphatization process. The sample and $NaPO_2H_2 \cdot H_2O$ were then annealed at 350 °C for 2, 3 or 4 h with a ramp heating rate of 2 $°C \cdot min^{-1}$ under an argon atmosphere to obtain the RuP/Ru@CNS catalysts. The samples were washed with ethanol and hydrochloric acid under ultrasonic conditions for 30 minutes to remove impurities. They were again washed with ultrapure water until the pH reached 7 and finally

dried at 60 °C for 24 h. The 3 h RuP/Ru@CNS phosphatization exhibited the best HER performance, and this sample was chosen for further study in this paper.

Synthesis of Ru@CNS.

Ru@CNS was prepared by hydrogen reduction of RuO_2@CNS at 350 °C for 3 h under a H_2 (5%)/Ar atmosphere.

Synthesis of RuP@CNS.

The RuP@CNS sample was obtained by vapor phosphatization of RuO_2@CNS. The RuO_2@CNS was heated to 500°C for 2 h in argon. Post-process cleaning was then the same as in the synthesis of RuP/Ru@CNS.

Characterization.

Powder XRD analysis was carried out using a Cu Kα radiation source with a 2θ range of 5 to 90° in order to identify the crystal phase of the samples. X-ray photoelectron spectroscopy (XPS) was conducted on an ESCALab250 electron spectrometer with a monochromatic 150 W Al Kα radiation source to confirm the composition of the sample. Field emission scanning electron microscopy (FESEM) was performed to obtain the morphology and elemental distribution of as-synthesized samples (JSM-7001F, JEOL, Tokyo, Japan). Imaging of the morphology and structure of prepared samples was conducted using a Scanning Transmission Electron Microscope (STEM, Thermo Fisher Scientific, G2 80–200) equipped with an energy-dispersive X–ray spectroscopy (EDS) detector and a high angle annular dark field (HAADF) detector operated at 200 kV. Elemental maps were obtained by EDS mapping in STEM mode. To prepare TEM samples, materials were dispersed in absolute ethanol by ultrasonication for 10 min and then drop–casted onto holey carbon coated copper TEM grids (Agar Scientific). X-ray absorption spectra (XAS) were acquired at the National Synchrotron Radiation Research Center (NSRRC), Hsinchu, Taiwan, ROC, and hard X-ray absorption data for the Ru K-edge

was collected at beamline BL17C.

Electrochemical measurements.

All electrochemical measurements were performed with a CHI 760E electrochemical test instrument in a standard three-electrode setup. We used Ag/AgCl as the reference electrode, a graphite rod as the counter electrode and a glassy carbon electrode (GCE; diameter 3 mm) coated with catalysts as the working electrode. The working electrode was fabricated as follows: 0.5 mg of as-synthesized sample was dispersed in a 300 μL mixture of 135 μL ultrapure water, 135 μL of ethanol and 30 μL 5% Nafion solution. Then mixture was sonicated to generate a homogeneous ink. Subsequently, 300 μL of ink was uniformly coated on 1 cm^2 of carbon fiber paper with a loading of 0.5 $mg·cm^{-2}$. 20.0% Pt/C was used as a standard reference sample with a loading of 1.0 $mg·cm^{-2}$. To evaluate the HER performance, we tested synthesized catalysts under acidic (0.5 M H_2SO_4), alkaline (1 M KOH) and neutral (1 M PBS) conditions at room temperature. All potentials were corrected according to the Nernst equation:

$$E_{RHE} = E_{Ag/AgCl} + 0.197 - 0.059pH - IR$$

Linear sweep voltammetry (LSV) was performed at a scan rate of 5 $mV·s^{-1}$ in all electrolytes. Cyclic voltammetry (CV) curves were acquired at a scan rate of 50 $mV·s^{-1}$ at a voltage of 0.0 ~ 0.2 V vs. RHE. Electrochemical impedance spectroscopy (EIS) was performed at different potentials and in a frequency range of 0.01 ~ 10 000 Hz. To measure the stability of the catalysts, CV scanning was repeated for 1000 cycles, and time-dependent current density was obtained without IR-correction. To evaluate the intrinsic activity of the as-synthesized catalyst, we calculated the electrochemical active surface area (ECSA) by electrochemical double-layer capacitance (C_{dl}). C_{dl} measurements were made from cyclic voltammetry curves with scanning rates of 20, 40, 60, 80, 100 and 120 $mV·s^{-1}$.

The AEM electrolytic cell was composed of a titanium plate, the catalyst and an anion exchange membrane. Firstly, RuO_2 and RuP/Ru@CNS were assembled into AEM electrolyzers

as anode and cathode respectively with anion exchange films. Then, 1.0 M KOH was injected into the positive electrode and negative electrode at the same time. Polarization curves were subsequently measured by LSV at a scan rate of 0.1 V s^{-1}.

DFT calculations.

DFT calculations were performed with VASP[1]. The Perdew-Burke-Ernzerhof (PBE) functional was used to treat exchange-correlation interactions[2]. For structure relaxation and calculation of electronic properties, the plane wave basis set was used with a kinetic energy cutoff of 500 eV, an energy convergence criterion of 10^{-5} eV and a (2×2×1) Monkhorst-Pack k-point sampling. The CI-NEB method was adopted to search for the minimum energy paths of the H_2O dissociation reaction[3, 4].

The Gibbs free energy was calculated using the following equation[5]:

$$\Delta G = \Delta E + \Delta E_{ZPE} - T\Delta S$$

where ΔE is the adsorption energy of adsorbed species and T is temperature (300 K). ΔE_{ZPE} and ΔS are the differences in zero-point energy and entropy, respectively.

It is well accepted that the reaction process for HER under alkaline conditions is:

(1) H_2O + e- + cat → H*-cat + OH^- (Volmer step)

(2) 2H*-cat → H_2 (Tafel step)

(3) H*-cat + H_2O + e- → cat + OH^- + H_2 (Heyrovsky step).

Results and Discussion

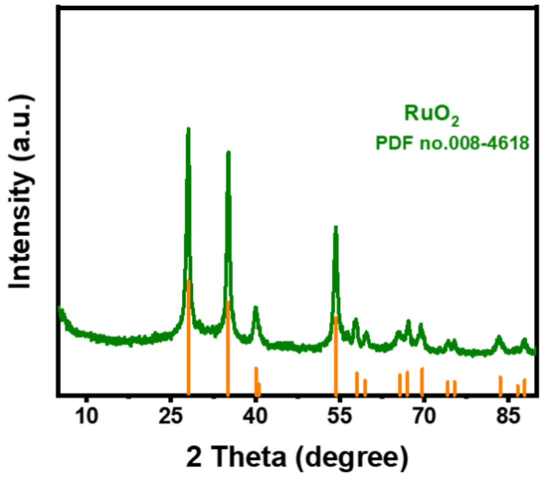

Figure S1. XRD pattern of the RuO_2@CNS.

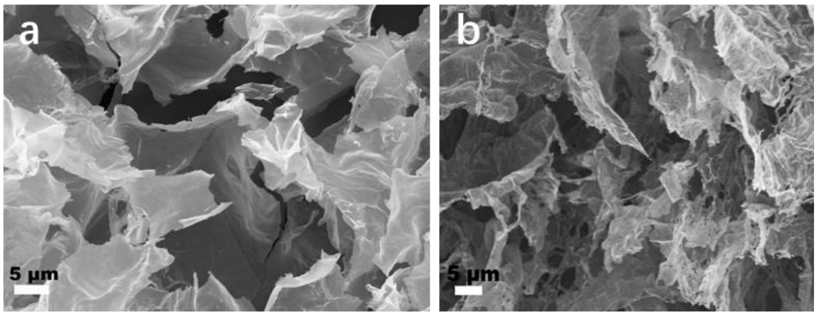

Figure S2. FESEM images of (a) Ru@CNS and (b) RuP@CNS.

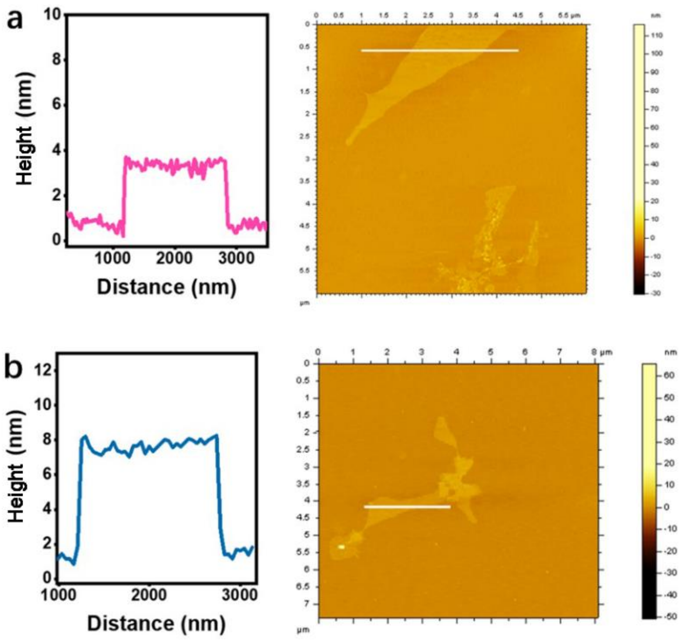

Figure S3. AFM images and the corresponding height profiles of (a) Ru@CNS and (b) RuP@CNS.

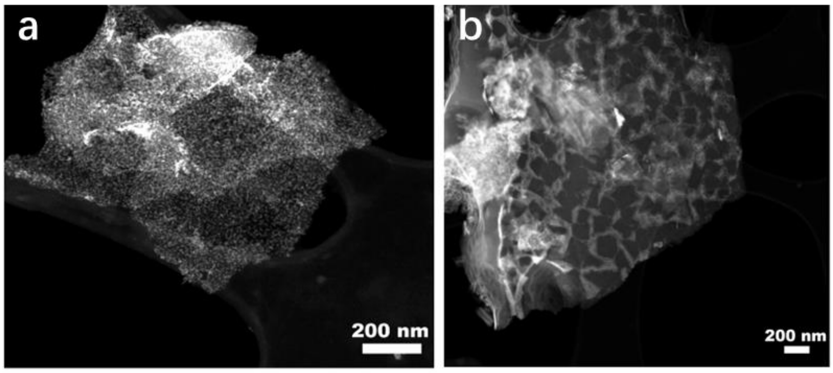

Figure S4. HAADF-STEM images of (a) Ru@CNS and (b) RuP@CNS.

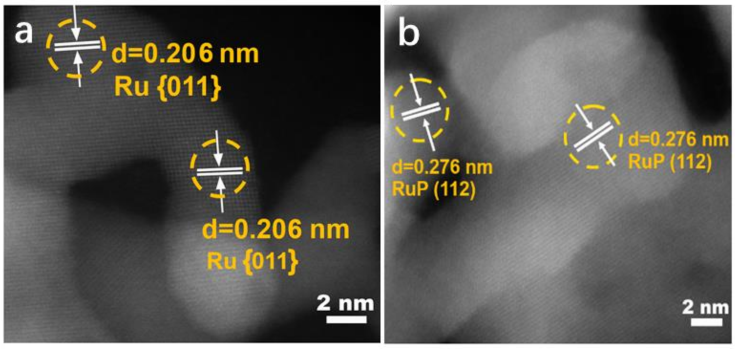

Figure S5. High-resolution HAADF-STEM image of (a) Ru@CNS and (b) RuP@CNS.

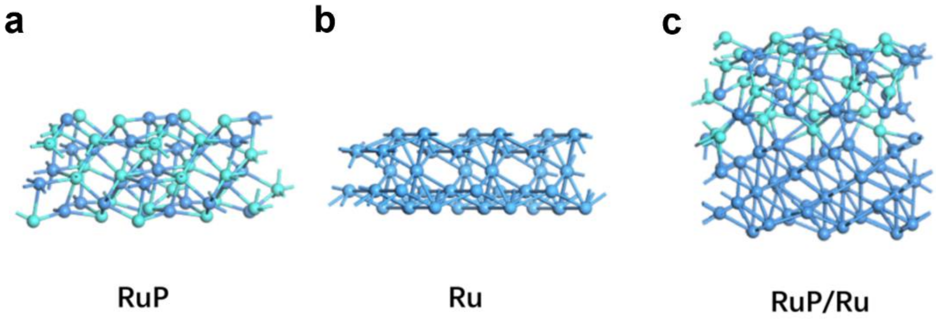

Figure S6. The optimized structure models of (a) pure Ru, (b) pure RuP, and (c) RuP/Ru heterostructure.

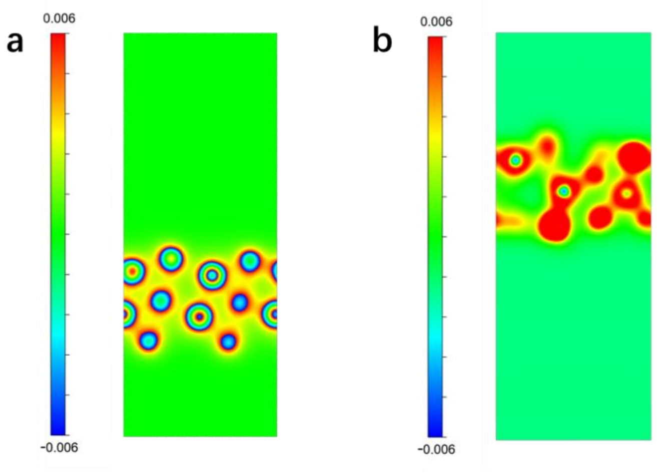

Figure S7. The calculated charge densities of (a) Ru and (b) RuP.

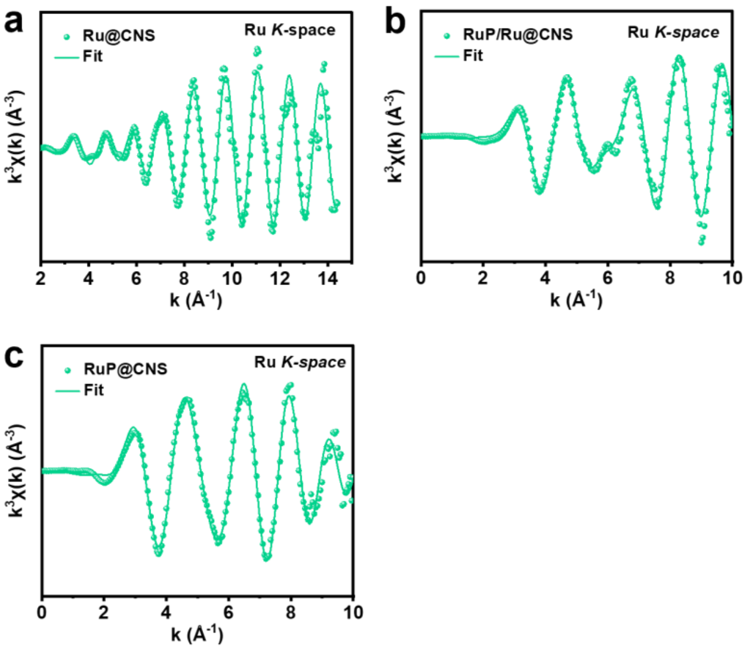

Figure S8. The best-fit EXAFS spectra in k space for (a) Ru@CNS, (b) RuP/Ru@CNS, and (c) RuP@CNS.

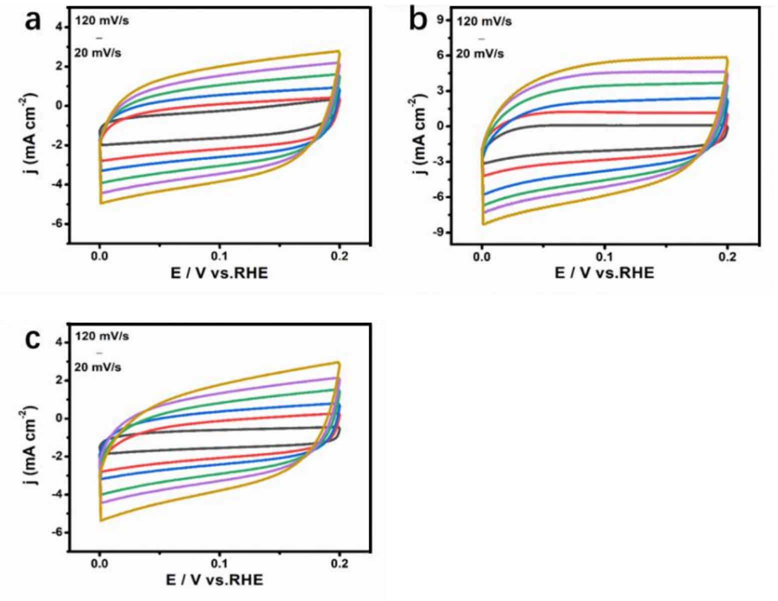

Figure S9. CV curves at different scan rate from 20 to 120 mV·s^{-1} of (a) Ru@CNS, (b) RuP/Ru@CNS, and (c) RuP@CNS in 1.0 M KOH electrolyte.

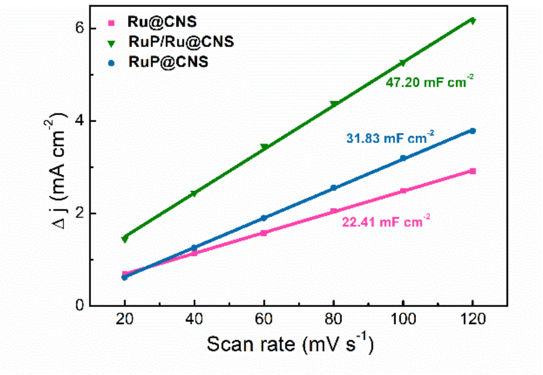

Figure S10. C_{dl} values for Ru@CNS, RuP/Ru@CNS and RuP@CNS in 1.0 M KOH electrolyte.

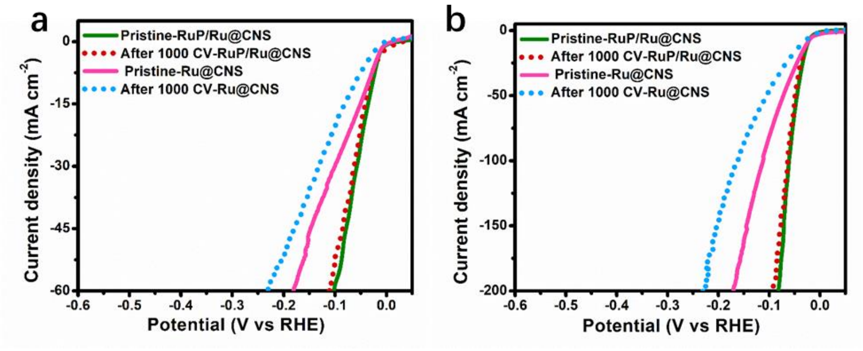

Figure S11. Cycling stability of RuP/Ru@CNS and Ru@CNS in (a) 1.0 M PBS electrolyte and (b) 0.5 M H_2SO_4 electrolyte.

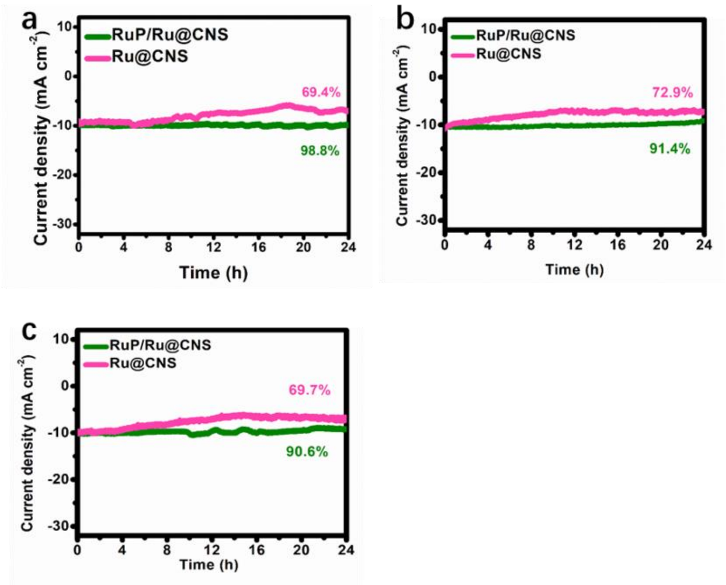

Figure S12. The time dependence of current density curves of RuP/Ru@CNS and Ru@CNS at static overpotentials of 10 $mA·cm^{-2}$ for 24 h in (a) 1.0 M KOH, (b) 1.0 M PBS and (c) 0.5 M H_2SO_4 electrolytes.

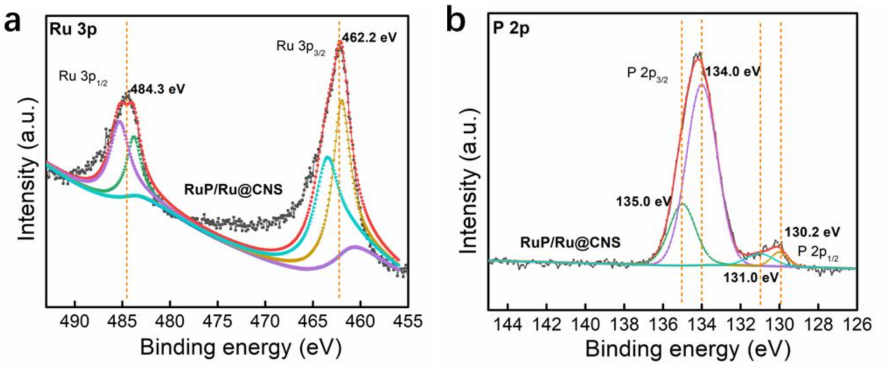

Figure S13. High resolution XPS spectra of (a) Ru 3p and (b) P 2p of RuP/Ru@CNS after 24 h stability test in 1.0 M KOH electrolyte.

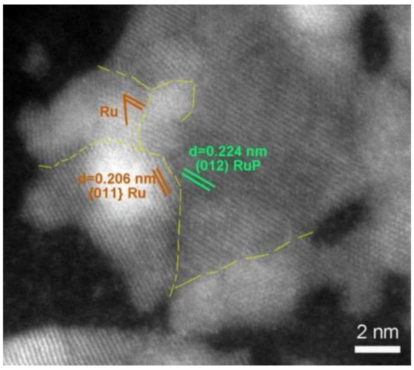

Figure S14. High-resolution HAADF-STEM image of the RuP/Ru@CNS after 24 h stability test in 1.0 M KOH electrolyte.

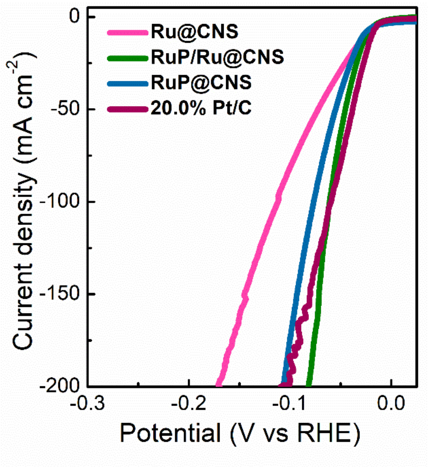

Figure S15. *IR*-corrected HER LSV curves Ru@CNS, RuP/Ru@CNS, RuP@CNS and 20% Pt/C in 0.5 M H_2SO_4 electrolyte.

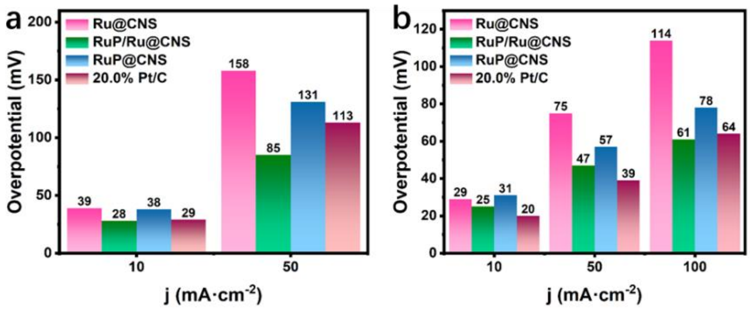

Figure S16. Comparison of overpotential of Ru@CNS, RuP/Ru@CNS, RuP@CNS in (a) 1.0 M PBS electrolyte and (b) 0.5 M H_2SO_4 electrolyte.

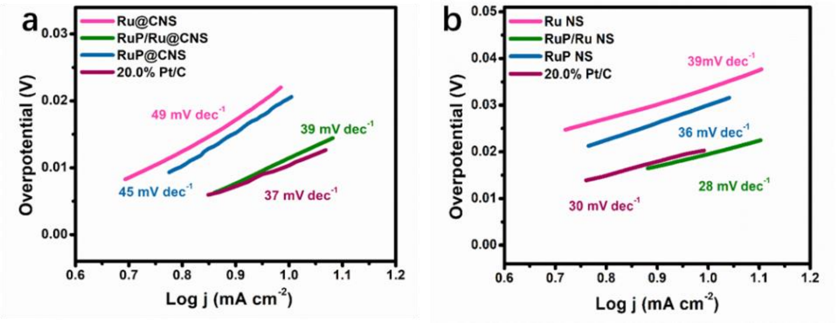

Figure S17. Tafel plots of RuP/Ru@CNS, RuP/Ru@CNS RuP@CNS and 20% Pt/C in (a) 1.0 M PBS electrolyte and (b) 0.5 M H_2SO_4 electrolytes.

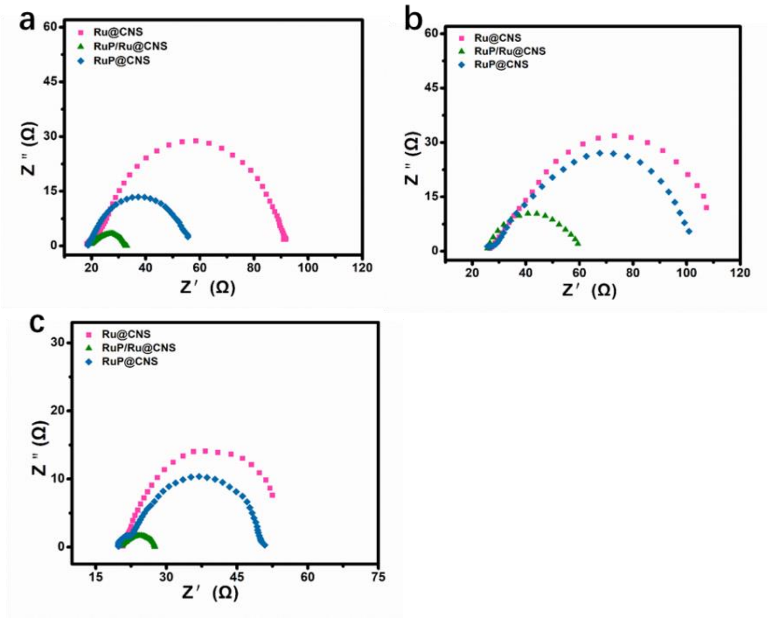

Figure S18. Nyquist plots at overpotential of 100 mV in (a) 1.0 M KOH, (b) 1.0 M PBS and c 0.5 M H_2SO_4 electrolytes.

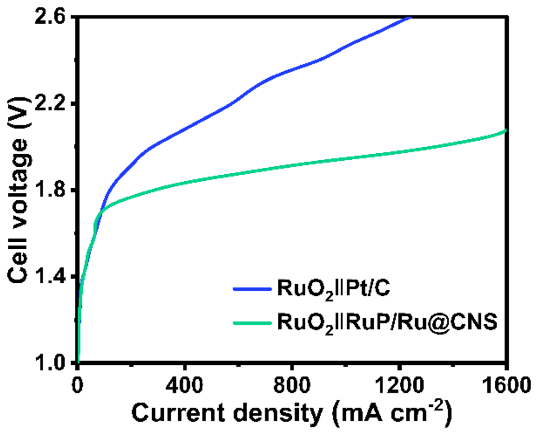

Figure S19. RuP/Ru@CNS Polarization curve detected in an AEM electrolyzer.

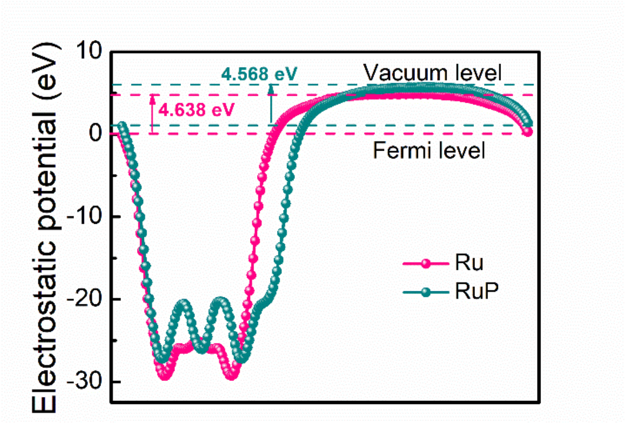

Figure S20. Work function estimation of Ru and RuP.

References

[1] G. Kresse, J. Furthmüller, *Phys. Rev. B* **1996**, *54*, 11169.

[2] J. P. Perdew, K. Burke, M. Ernzerhof, *Phys. Rev. Lett.* **1996**, *77*, 3865.

[3] G. Henkelman, H. Jónsson, *J. Chem. Phys.* **2000**, *113*, 9978.

[4] G. Henkelman, B. P. Uberuaga, H. Jónsson, *J. Chem. Phys.* **2000**, *113*, 9901.

[5] J. K. Nørskov, J. Rossmeisl, A. Logadottir, L. Lindqvist, J.R. Kitchin, T. Bligaard, H. Jonsson, *J. Phys. Chem. B* **2004**, *108*, 17886.

Author Contributions

D. Li designed and performed the experiments. J. Ren performed the theoretical calculations. D. Zheng performed some experiments and characterization. R. Cai and S. Haigh conducted aberration-corrected high-resolution transmission electron microscope measurement. C. Dong and Y. Huang performed X-ray adsorption measurement. F. Gong discussed the theoretical calculations. D. Li, X. Liu, J. Liu, Y. Liu and D. Yang wrote the manuscript with input from all authors. All authors discussed results and conclusions.
